# Genomic Diversity of Two Hydrocarbon-Degrading and Plant Growth-Promoting *Pseudomonas* Species Isolated from the Oil Field of Bóbrka (Poland)

**DOI:** 10.3390/genes10060443

**Published:** 2019-06-11

**Authors:** Valeria Imperato, Miguel Portillo-Estrada, Breanne M. McAmmond, Yorben Douwen, Jonathan D. Van Hamme, Stanislaw W. Gawronski, Jaco Vangronsveld, Sofie Thijs

**Affiliations:** 1 Environmental Biology, Centre for Environmental Sciences, Hasselt University, Agoralaan Building D, 3590 Diepenbeek, Belgium; valeria.imperato@uhasselt.be (V.I.); d_yorben@hotmail.com (Y.D.); jaco.vangronsveld@uhasselt.be (Y.V.); 2 Centre of Excellence PLECO, Department of Biology, University of Antwerp, Universiteitsplein 1, B-2610 Wilrijk, Belgium; Miguel.PortilloEstrada@uantwerpen.be; 3 Department of Biology Sciences, Thompson Rivers University, 805 TRU Way, Kamloops, BC V2C 0C8, Canada; Breannemcammond95@gmail.com (B.M.M.); Jvanhamme@tru.ca (J.D.V.H.); 4 Warsaw University of Life Sciences, Faculty of Horticulture, Biotechnology and Landscape Architecture, Nowoursynowska Road 159, 02-776 Warsaw, Poland; stanislaw_gawronski@sggw.pl; 5 Department of Plant Physiology, Faculty of Biology and Biotechnology, Maria Skłodowska-Curie University, 20-950 Lublin, Poland

**Keywords:** naphthalene, toluene, hydrocarbons, plant growth promotion, bioremediation, *Pseudomonas*, soil pollution, phytoremediation

## Abstract

Hydrocarbon-degrading bacteria are important resources for use in phytoremediation applications. Yet, for many hydrocarbonoclastic strains the genetic information regarding pollutant degradation and detoxification has not been thoroughly revealed. In this study, hydrocarbon-degrading bacteria were isolated from a long-term oil-polluted soil in Bóbrka, Poland. *Pseudomonas* spp. was the most dominant species. Of all 69 isolated strains tested in the laboratory using qualitative biochemical assays, 61% showed the capability to use diesel as sole carbon source, 33% could produce indole, 19% produced siderophores, 36% produced organic acids, and 54% were capable of producing 1-aminocyclopropane-1-carboxylate (ACC)-deaminase. From all morphologically and genetically different strains, two representative *Pseudomonas* spp., strain VI4.1 and VI4T1, were selected for genome sequencing. Genomic analyses indicated the presence of the full naphthalene dioxygenase operon (plasmid and chromosomal), of genes involved in the degradation of BTEX compounds (Benzene, Toluene, Ethylbenzene, Xylene) and alkanes (*alkB* gene) as well as the anthranilate degradation pathway (strain VI4T1) and terephthalate dioxygenase protein (strain VI4.1). Proton transfer reaction time-of-flight mass spectrometry (PTR-TOF-MS) analyses confirmed naphthalene and BTEX degradation within seven days. Motility, resistance to abiotic stresses, high and low temperatures, low pH, and salinity were confirmed at the genetic level and experimentally verified. The presence of multiple degradative and plant growth promotion genes, together with the in vitro experimental evidence, indicates the high value of these two strains and their potential use for sustainable site clean-up.

## 1. Introduction

Environmental spills with recalcitrant pollutants such as mono- and polycyclic aromatic hydrocarbons (MAHs, PAHs) are a problem worldwide, threatening the environment and human health [1]. Many of the diverse group of aromatic hydrocarbons are classified as priority pollutants by the US Environmental Protection Agency [2]. Naphthalene has also been classified as a group C possible human carcinogen. When oil spill accidents occur on land, degradation of petroleum hydrocarbons by indigenous microorganisms is often a slower process to remediate contaminated areas in comparison to traditional physical and chemical remediation treatments, often due to low microbial numbers and activity [3]. Microbial bioremediation, the process of pollutant degradation by microorganisms, is a green, cheap, and safe approach to cleaning up polluted sites [4,5]. This process can be improved by promoting the growth of endogenous bacteria in the polluted soil itself (biostimulation) or by introducing hydrocarbon-degrading bacteria (bioaugmentation) [6,7]. Frequently, due to strict legislations that impede the introduction of non-indigenous microorganisms at the site of interest, bioaugmentation is implemented by the use of native strains, or strains which carry the degradative genes on plasmids which can be transferred to the indigenous population though means of natural gene transfer. Studies demonstrated how this approach is considered the best choice for bio-detoxification of soils with a low degradation potential by the indigenous bacterial communities [8,9]. 

Undoubtedly, it is a challenge to find an effective and efficient method to remediate polluted soils [10], especially because of the complexity and specificity of each site. Oil spillages cause profound and persistent changes in soil properties. pH may vary, as well as nutrient availability (e.g., carbon, nitrogen), with high concentrations of mono- and polycyclic aromatic hydrocarbons, often in combination with heavy metal pollution (nickel, lead, chromium, copper, zinc, cadmium). All these factors affect and re-shape the structure of indigenous microbial communities [11,12]. In this highly complex framework, the study and isolation of novel native ring hydroxylating bacterial strains constitutes an approach with high potential and a powerful alternative to the traditional physical and chemical remediation approaches.

In this study we combined traditional microbiological methods to isolate and characterize potential degraders from an ancient oilfield, combined with a bacterial genomic investigation aiming to reveal the genetic backgrounds of two interesting hydrocarbon-degrading bacteria belonging to the genus *Pseudomonas*. Our study aims to elucidate (1) the degradation pathways of linear and aromatic hydrocarbons, (2) plant growth promotion traits (hormones and stress enzyme reduction), and (3) the adaptation of the two identified *Pseudomonas* spp. to life in soil, tolerance to various stresses, and extraordinary degradation capacities of micropollutants.

## 2. Materials and Methods 

### 2.1. Isolation of Hydrocarbon-Degrading Bacteria Using a Sandwich Diffusion System

**Figure 1:**
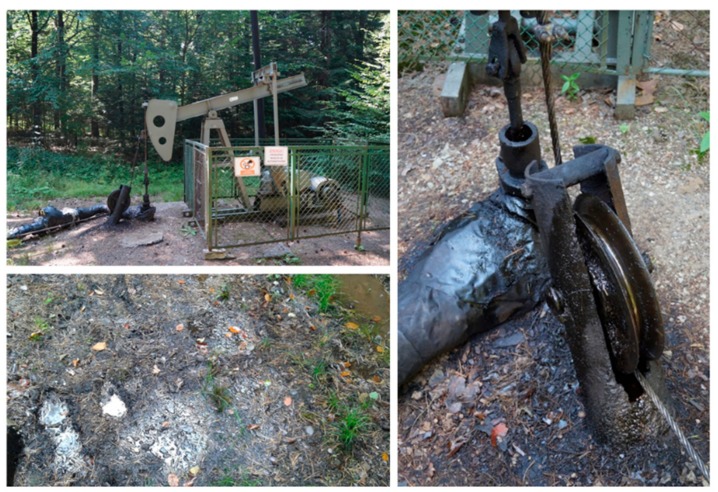
Sampling site of Bóbrka with spilled black crude oil.

In June 2016, we sampled the historical oilfield forest soil in Bóbrka, Poland (Latitude: 49.616449; Longitude: 21.710454; Altitude: 350.45 m). The Bóbrka sampling site is unique in the world since it has one of the oldest oil wells still in production since 1854, and still pumps one barrel a day. The black top soil layer near an oil pump (Figure 1) was sampled with a sterilized shovel and stored in self-sealed bags. These bags were kept in temperature-controlled boxes and shipped to Hasselt University.

In the lab, one gram of soil was added into 15-mL sterile falcon tubes containing 5 mL of 0.1 M phosphate-buffered saline (PBS, pH = 7.0). After vortexing, tubes were incubated for one hour on an orbital shaker at 120 rpm to allow the microorganisms to effectively being released from the soil particles. In the meantime, Teflon components, steel screws, and hydrophilic membranes of a custom-made sandwich diffusion system (Figure S1) were sterilized by autoclaving (120 psi, 30 min). Each sterilized “perforated plate” was dipped into a mixture of Bushnell Haas medium [13] with 0.7% w/v Gelzan (G1910; Sigma-Aldrich, St. Louis, Missouri, USA) supplied with 0.1% CaCl_2_ and the soil dilution to obtain a 10^-5^ concentration of the soil suspension. This dilution was chosen to obtain the growth of one or few bacterial cells per well. The mixture was left to solidify under a sterile laminar flow after which the membranes were placed on both sides of the agar-filled plates (WHA111103; Nucleopore® polycarbonate track-etched membrane; 47-mm diameter, 0.05-μm pore size, Whatman, Maidstone, UK). The membranes allow the exchange of nutrients and molecules from the soil to the water-agar containing bacterial cells but not the passage of bacterial cells. Finally, the sandwich diffusion systems were closed with screws and buried into the wetted soil and incubated at 20 °C for one month. 

After one month of incubation, the sandwich systems were disassembled and each agar plug was pushed out with sterile toothpicks into a sterile deep-well masterblock filled with Bushnell Haas medium and incubated overnight at 30 °C on an orbital shaker. The next day, 100 μL of each bacterial suspension were plated onto 1/10 869 rich medium [14] supplemented with 2 mM indole according to Nagayama et al. 2015 [15]. After incubation for seven days at 30 °C, the colonies producing a dark-blue pigment were selected as positive for aromatic compound ring hydroxylase activity (conversion of the colourless indigo to dark-blue indole by dioxygenase enzymes) [15]. *Pseudomonas putida* f1 [16] and *Pseudomonas aeruginosa* WatG [17] were used as positive controls.

### 2.2. Growth Conditions and Genotypic Characterization of the Bacterial Strains 

Bacteria stored in a 96 well masterblock (Greiner Bio-One, Kremsmünster, Austria) and those scoring positive on the indole test were grown in 5 mL of 869 medium [14] and incubated for 24 h at 30 °C while shaking at 200 rpm. Two mL of culture broth were used for total DNA extraction using the Qiagen Blood and Tissue kit (Qiagen, Venlo, Netherlands). The quality of the purified genomic DNA was checked using a NanoDrop 1000 UV-Vis spectrophotometer (Thermo Fisher Scientific, Wilmington, DE, USA). The 27F (5′ AGAGTTTGATCMTGGCTCAG 3′) and 1492R (5′ TACGGYTACCTTGTTACGACTT 3′) primers were used for the amplification of the 16S rRNA gene [18]. The PCR master mix consisted of: DNA template (10 ng μL^−1^), 1 × High Fidelity PCR buffer (Invitrogen, Carlsbad, CA, USA), 0.2 mM dNTPs, 2 mM MgCl_2_, 0.2 μM each of the forward and reverse primers, and 1 U High Fidelity Platinum Taq DNA polymerase (Invitrogen, Carlsbad, CA, USA) per 50 μL. PCR conditions were set as follow: denaturation at 94 °C for 5 min, 30 cycles of 94 °C for 1 min, 54 °C for 45 s, and 72 °C for 1.5 min, followed by a final extension of 10 min at 72 °C. Confirmation of PCR product amplification was tested by running a 1% agarose gel electrophoresis. The PCR products for each strain were sent to Macrogen (Amsterdam, The Netherlands) for 16S rRNA gene Sanger sequencing. Sequences were quality checked using Geneious v4.8.5 (Biomatters ApS, Aarhus, Denmark) and blasted against the nucleotide sequences present in the Ribosomal Database Project (RDP Release 11).

### 2.3. BOX-Fingerprinting of Potential Hydrocarbon Degraders and nahAc PCR Amplification

BOX-fingerprinting was performed on the dark blue pure colonies from the indole test. The BOX_A1R primer (5′-CTACGGCAAGGCGACGCTGACG-3′), as described by Rademaker (1997), was used in the PCR reaction mixture described above. The reaction started with denaturation for 10 min at 95 °C, followed by 30 cycles of denaturation for 1 min at 95 °C, annealing for 1 min at 50 °C, and elongation for 8 min at 68 °C, with the final elongation for 8 min at 68 °C. PCR products were visualized on a 1.5% agarose gel stained with gel-red [19]. 

Naphthalene dioxygenase Fe-S large subunit (NahAc) genes were amplified with the primers nah-F (5′ CAAAARCACCTGATTYATGG 3′) and nah-R (5′ AYRCGRGSGACTTCTTTCAA 3′) using PCR conditions as described by Baldwin et al. 2003 [20]. PCR products of the correct size were purified and sent to Macrogen (Amsterdam, The Netherlands) for Sanger sequencing. Multiple sequence alignment was performed with Clustal Omega.

### 2.4. Genome Sequencing and Assembly

RNA‑free DNA was extracted from stationary phase cells of strains *Pseudomonas* sp. VI4.1 and *Pseudomonas veronii* VI4.1 grown in 869 rich broth prior to digesting and ligating sequencing adaptors and barcodes using an Ion Xpress Plus Fragment Library Kit (Life Technologies Inc., Burlington, ON, Canada). Adaptor‑ligated DNA was size-selected to 480 bp on a 2% E‑Gel SizeSelect agarose gel, and Agencourt MAPure XP beads (Beckman Coulter, Mississauga, ON, Canada) were used for purification. The library dilution factor was determined using an Ion Library Quantitation Kit prior to amplification and enrichment with an Ion PGM Template Hi‑Q OT2 kit on an Ion OneTouch 2 system. The enriched Ion Sphere Particles were quantified using an Ion Sphere Quality Control Kit prior to sequencing on a 316v2 chip with an Ion PGM Hi‑Q View Sequencing Kit on an Ion Torrent PGM (Life Technologies Inc., Carlsbad, CA, USA).

Sequencing of strain VI4T1 generated a total of 1.1 million reads (mean length 300 bases, or 331 Mb of data (>307 M Q20 bases) in Torrent Suite 5.0.5. For strain VI4.1, 1.24 million reads (mean length 299 bases) generated 371 Mb data (>344 M Q20 bases). Reads were assembled using SPAdes 3.11.1 (uniform coverage mode; kmers 21, 33, 55, 77, 99, 127) into contigs greater than 1000 bp [21]. The assembly of strain VI4T1 resulted in 211 contigs with consensus length of 7,150,343 bp (>1000 bp) at 27x coverage (60.54% G+C content, largest contig of 237,158 bp; and N50 = 68,401 bp). Assembly of VI4.1 resulted in 185 contigs with a consensus length of 7,346,306 bp at 26.0X coverage (60.02% G+C content; largest contig of 285,953 bp; N50=73,645 bp). Plasmids were predicted using plasmidSPAdes, with kmers 21, 33, 55, 77, 99, 127 [22].

### 2.5. Genome Annotation and Phylogenetic Tree of the 16S rRNA Gene Sequences

Open Reading Frame (ORF) prediction and gene annotation were performed using the RAST annotation system (Overbeek et al. 2014) [23], Prokaryotic Genome Automatic Annotation Pipeline (PGAP) of the National Center for Biotechnology Information (NCBI) [24] and the platform MicroScope using the tool Magnifying Genomes, MaGe (http://www.genoscope.cns.fr) [25]. Clusters of Orthologous Genes (COGs) [26] and metabolic pathway reconstruction was carried out using the databases of Kyoto Encyclopedia of Genes and Genomes (KEGG) [27] and MetaCyc [28].

Comparative genome analyses were performed in SimpleSynteny, which uses NCBI BLAST, BioRuby and RMagick to map genes onto genomes and generate figures [29]. Default settings were used with BLAST E-value of 0.001 and 50% minimum query coverage.

The in silico DNA–DNA hybridisation (DDH) values between VI4.1 and VI4T1 and closely related strains was calculated using the online Genome-to-Genome distance calculator version 2.1 (GGDC) (http://ggdc.dsmz.de/ggdc_background.php#) [30,31]. Circular maps of the chromosomes and plasmids were generated in MaGe and in Bandage version 0.8.0 [32]. Clustered regularly interspaced short palindromic repeats (CRISPRs) were identified using the web service (http://crispr.u-psud.fr/Server/CRISPRfinder.php).

The bioinformatics prediction of genes associated with the biodegradation of naphthalene, BTEX (Benzene, Toluene, Ethylbenzene, Xylene), and aliphatic hydrocarbons was based on Basic Local Alignment Search Tool (BLAST) searches, Protein family (PFAM) queries and conserved domain searches, using the databases MetaCyc, UniProt database (http://www.uniprot.org/) and NCBI. Visualisations of the plasmid and operon structures was performed with SnapGene version 3.2.1.

A phylogenetic tree with VI4.1, VI4T1, and closely related strains evaluated against the Ribosomal Database was built in MEGA7 [33]. The evolutionary distances were computed using the Maximum Composite Likelihood method [34].

### 2.6. Biochemical and Chemotaxonomic Identification

The biochemical metabolite profile of VI4T1 and VI4.1 was determined using Gen-III MicroPlates (Biolog, Hayward, USA). Bacteria were grown in 869 rich medium overnight, subsequently resuspended in sterile 10 mM sodium phosphate buffer (pH 7.2) to an Optical Density (OD) of one checked at 600 nm. Next day, 100 μL of each strain was inoculated into each well of the Gen-III microplate and incubated at 30 °C for five days. Each microplate allows to carry out 94 phenotypic tests: 71 carbon source utilization assays and 23 chemical sensitivity assays. Each well contains a redox tetrazolium dye that changes colour as a result of cellular respiration providing a reliable metabolic fingerprint, which can be evaluated by measuring absorbance at 595 nm.

In addition, a GC Analysis of total fatty acid methyl esters (FAMEs) was performed at EMSL Analytical (Cinnaminson, NJ, USA) to identify the strains based on the FAME fingerprint and using the Sherlock Microbial Identification system (MIS).

### 2.7. In Vitro Estimation of Diesel Oil Degradation Capabilities

The bacterial strains were tested in triplicates for their capability to use diesel as the only carbon source using the 2,6-dichlorophenol indophenol (DCPIP) assay [35]. DCPIP is a compound with high affinity for electrons which when oxidized by metabolic reactions turns from blue to colourless. In brief, bacteria were pre-cultured in 5 mL of 869 rich medium at 30 °C and 160 rpm on an orbital shaker until OD_660_ nm = 1.0. Cells were pelleted by centrifugation (4000 g for 20 min), washed three times with 10 mM MgSO_4_ and incubated overnight at 30 °C to allow the bacteria to use all remaining carbon source traces (starvation). Subsequently, 750 μL of Bushnell and Haas medium supplemented with 50 μL DCPIP solution (100 μg ml^−1^) were added to a 2-mL sterile microcentrifuge tube. Subsequently, 80 μL of cell suspension and 5 μL filter sterilized diesel were added. Cells were cultivated in the dark to avoid photodegradation of the redox dye (30 °C; 120 rpm) for 1 week. The colour of the reaction medium was compared with three controls: two negative controls with respectively no diesel and no bacteria and a positive control with *Pseudomonas aeruginosa* WatG strain [17]. Strains were scored as positive for microbial hydrocarbon degradation capability if the solution looked clear, and negative if it persisted in blue.

### 2.8. Analysis of Naphthalene Degradation Using High-Resolution Proton Transfer Reaction Time-of-Flight Mass Spectrometry (PTR-TOF-MS)

The capabilities of the strains to use naphthalene as sole carbon source was assessed by measuring naphthalene degradation using PTR-TOF-MS. 20 mL sterile glass vials with 1.5 g of sterilised sand were inoculated with 200 µL of bacteria suspension (O.D.=1) in Bushnell Haas medium without carbon source. Vials were spiked with 0.3 µL of naphthalene against the inner glass wall to saturate the head-space and incubated for 1 week at 25 °C. A set of vials with the same conditions but no bacteria was used as a control. All experiments were carried out in triplicate.

For PTR-TOF-MS analyses, the headspace of the vials was sampled through the Teflon septum with a 50-mL glass syringe (Z314587, Fortuna Optima, Sigma-Aldrich) equipped with a three-way stopcock attached to the syringe Luer lock tip. The sample was injected into the air inlet of the PTR-TOF-MS instrument (PTR-TOF 8000, Ionicon, Innsbruck, Austria). The instrument drift tube was operated at a field density ratio (E/N) of ≈130 Td, resulting from 2.2 mbar pressure, 80 °C temperature, and 530 V of electric potential. In short, the air sample met a rich mixture of H_3_O^+^ that protonated the volatiles contained in the air sample. The reaction rate coefficient between the hydronium ion (H_3_O^+^) and naphthalene was assumed 2.45 × 10^−9^ cm^3^ s^−1^ [36].

### 2.9. In Vitro Plant Growth Promotion Activity of the Bacterial Strains 

Bacteria were screened in vitro for plant growth promoting traits including 1-aminocyclopropane-1-carboxylate (ACC)-deaminase, siderophore production, acetoin, organic acid, and indole-3-acetic acid (IAA) production, phytate mineralization and nitrogen fixation. The production of ACC-deaminase was estimated by monitoring the amount of α-ketobutyrate generated by the enzymatic hydrolysis of ACC [37]. Siderophore release was evaluated via Chrome Azurol S (CAS) assay [38]. The production of the phytohormone IAA was estimated by a colorimetric assay using Salkowski’s reagent [39]. The production of the volatile plant growth promoting compound acetoin was assessed using the Voges-Proskauer assay [40]. The evaluation of the results was based on the observations of the colorimetric reactions after 5 days of incubation at 30 °C. Phytate mineralization was evaluated after 12 days of incubation by observing the halo produced around the colonies growing on solid medium supplemented with Na-phytate [41]. Organic acids produced by plant-associated bacteria facilitate the solubility of nutrients in soil, thus facilitating the uptake of nutrients by the plant. The method of Cunningham and Kuiack was used to check their production [42]. Bacteria able to fixate nitrogen possess the enzyme nitrogenase which catalyses the reduction of atmospheric nitrogen to ammonium that can be detected by a colour change on semisolid medium without any nitrogen source [43]. 

### 2.10. *Pseudomona* VI4.1 and VI4T1 Motility

Bacterial motility was tested in sterile glass tubes previously filled with 869 semi-solid agar medium. Inoculation was performed with a sterile straight wire, making a single stab down the centre of the tube to about half the depth of the medium. Subsequently, the tubes were incubated at 30 °C and observed for bacterial growth at regular intervals (1 until 7 days). Non-motile bacteria generally grow into the stab-line, have sharply defined margins and leave the surrounding medium clearly transparent while motile bacteria typically grow by diffusing throughout the medium, rendering it slightly opaque. 

### 2.11. Sequence Database Accession Numbers

The genome sequencing projects have been deposited at DDBJ/EMBL/GenBank with strain VI4T1, Accession Number MULN00000000 and BioProject PRJNA369437, and for strain VI4.1, Accession Number NZ_MULM00000000 and BioProject PRJNA224116. The Genbank accession numbers for the sequenced naphthalene dioxygenase genes are MN030640-MN030657 with associated partial 16S rRNA gene sequences of the isolates: MN006582-MN006610. The other bacterial isolates with 16S rRNA gene sequence identification were also deposited in NCBI Genbank with accession numbers: MN030267-MN030332.

## 3. Results and Discussion

### 3.1. Genotypic and Functional Characterization of the Cultivable Hydrocarbon-Degrading Bacterial Community from the Oilfield of Bóbrka

**Figure 2:**
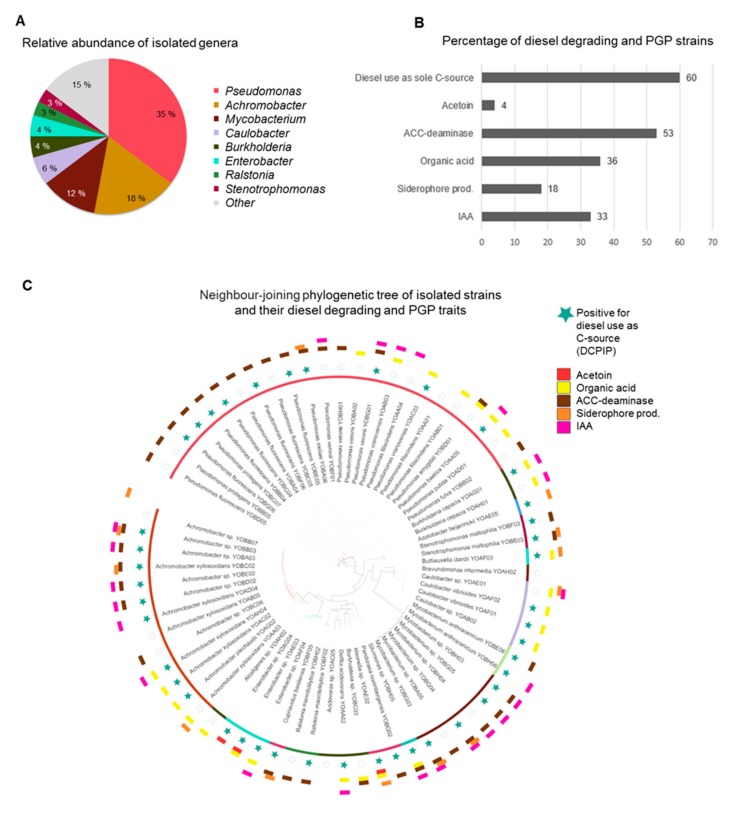
(**A**) Relative abundance of isolated oil tolerant bacteria using the diffusion sandwich system. (**B**) Percentage of strains displaying plant-growth promotion (PGP) properties, specifically Acetoin, Organic acid, 1-aminocyclopropane-1-carboxylate (ACC)-deaminase, siderophore and indole-3-acetic acid (IAA) productions. (**C**) neighbour-joining phylogenetic tree of all isolated taxa and their PGP and diesel-degrading properties. Coloured blocks and coloured stars indicate that the strain scored positive for the tests, non-coloured (empty) blocks and stars indicate the strain should not produce/utilize the compound evaluated by the corresponding assay.

In search for novel hydrocarbon and plant growth promoting strains, we performed an isolation and selective enrichment approach to identify and characterize novel microorganisms capable of degrading oil-related pollutants. Seventy bacterial strains were isolated by using the sandwich diffusion system which had incubated for one month in polluted soil from Bóbrka with crude oil (Figure 2A). 16S rRNA Sanger sequencing showed a dominance of *Pseudomonas* spp. (35%) followed by *Achromobacter* (18%), *Mycobacterium* (12%), *Caulobacter* (6%), *Burkholderia* (4%), *Enterobacter* (3%), *Ralstonia* (3%), and *Stenotrophomonas* spp. (3%) (Figure 2A). A high percentage (60%) of the strains were able to use diesel as sole carbon source. A majority of the strains produced ACC-deaminase (53%). ACC-deaminase is an important trait of many soil and plant-associated bacteria as they can facilitate growth and development of the plant by lowering stress ethylene levels [44]. 33% of the strains produced the hormone indole-3-acetic acid. Strains producing IAA can have a phyto-stimulation effect on the host plant or pathogenic, depending on the concentration [45]. Bacteria use this hormone to interact with the host plants as part of their colonization strategy and to circumvent the basal plant defence mechanisms. It can also serve as signalling molecule between bacteria. Siderophore production and organic acid production was present in 18% and 36% of the isolated strains, respectively (Figure 2B). This can aid in the solubilization of iron and phosphorous in soil, making it more available to microbial and plant uptake. Only 6% of the strains were scored as positive for acetoin production. This ketone showed to enhance plant growth by stimulating root development and increasing resistance against pathogens and drought stress. An overview of the traits per species is shown in Figure 2C. The PGP and diesel use as sole carbon source traits were diversely spread across the different taxa. Strains with multiple PGP traits are most interesting for in vivo plant growth promotion tests, such as *Pseudomonas veronii* YOB01, *Burkholderia cepacia* YOAG01, *Mycobacterium* YOBG04, and *Achromobacter* YOBB03, amongst others. 

### 3.2. BOX-Fingerprinting

**Figure 3:**
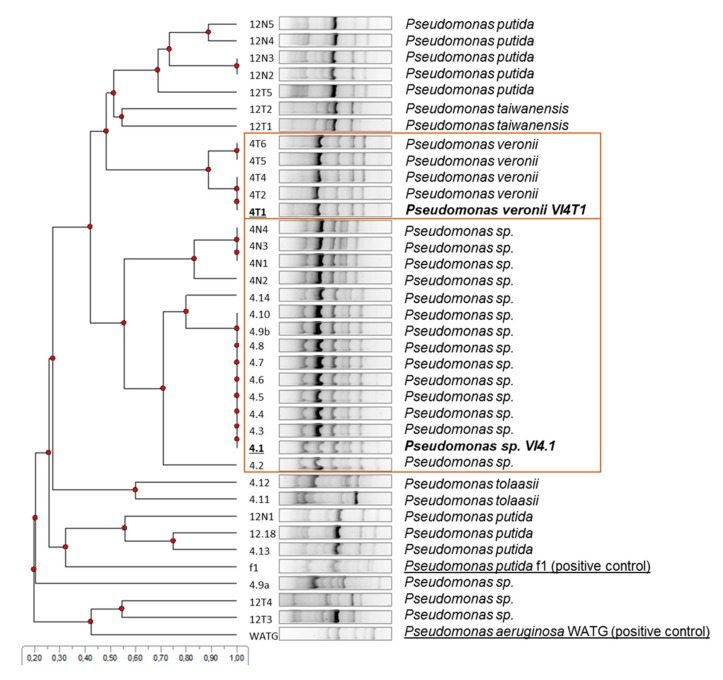
Cluster analyses of BOX-fingerprint PCR products of indole-positive isolates. Strains VI4.1 and VI4T1 were chosen as representative isolates with positive naphthalene dioxygenase large subunit (*nahAc*) gene PCR amplification. *Pseudomonas putida* f1 and *Pseudomonas aeruginosa* WatG were included as reference strains with naphthalene dioxygenase activity.

BOX-fingerprinting was performed on the isolates that scored positive on the indole/indigo test and thus possess dioxygenases that can have activity against naphthalene and toluene. Based on the differences in the fingerprint patterns and partial 16S rRNA gene identification, eight groups could be discriminated. Subsequently, those strains which gave a positive amplification band and blast hit to an aromatic compound dioxygenase were down-selected (in squares). A multiple sequence alignment of the Rieske subunit of the naphthalene dioxygenases showed a high similarity with the naphthalene dioxygenase large subunit (*nahAc*) of *Pseudomonas putida*, encoded on plasmid pAK5, and verified experimentally to use naphthalene (Figure S2). Based on this information, we chose to sequence two representative strains, one of the *Pseudomonas* sp. group (VI4.1) and *Pseudomonas veronii* group (VI4T1), Figure 3. 

### 3.3. General Features of the Draft Genomes of Strain VI4T1 and VI4.1

The 7,150,343-bp genome of strain VI4T1 (GC 60.54%) contains 7962 genes, of which 7809 are Coding Sequences (CDSs), 9 are rRNAs (7, 1, 1 for 5S, 16S, 23S), and 60 are tRNAs comprising 20 different tRNA types. The 7,346,306-bp genome of *Pseudomonas* sp. VI4.1 (GC 60.02%) contains 8082 genes, of which 7922 are CDS, 9 are rRNAs (7, 1, 1 for 5S, 16S, and 23S, respectively), and 58 are tRNAs composed of 21 different tRNA types. The MaGe server classified 73.65% and 74.38% of the CDSs in at least one Cluster of Orthologous Group (COG) for VI4T1 and VI4.1, respectively. In addition, 83.90% and 82.87% of the CDSs are classified in at least one Evolutionary genealogy of genes: Non-supervised Orthologous Groups (EGGNOG) for VI4T1 and VI4.1 (Figure S3). The most abundant EGGNOG categories for VI4T1 are, amino acid and transport, transcription, inorganic ion transport and metabolism, energy production, while for VI4.1, the most dominant categories were secondary metabolite biosynthesis, inorganic ion transport, lipid transport and metabolism, and coenzyme transport and metabolism (Figure S3). In strain VI4.1, 21 CRISPR sequences and nine transposases were found, while in VI4T1, seven CRISPRs, two Cas (CRISPR-associated), and 10 transposases were present. CRISPR, clustered regularly interspaced short palindromic repeats, along with Cas are a bacterial defence system against bacteriophage predation. This all indicates that both strains have experienced extensive and complex genetic alterations and acquired new genetic elements by bacteriophages and exchanges by lateral gene transfer.

**Figure 4:**
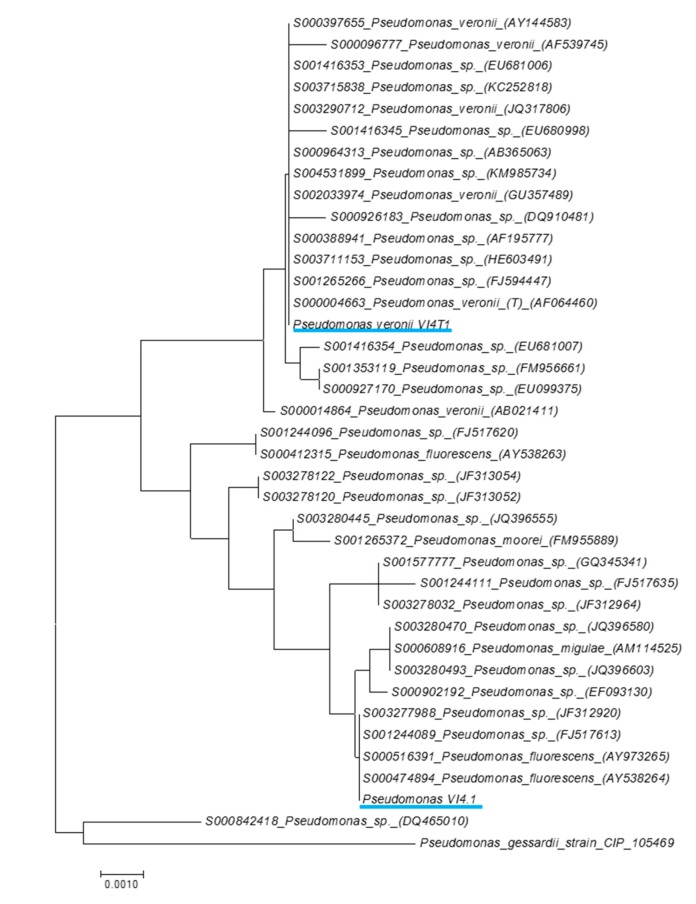
16S rDNA phylogenetic tree of *Pseudomonas veronii* VI4T1 and *Pseudomonas* sp. VI4.1 compared to closely-related strains and with *Pseudomonas gessardii* as an outgroup. Closely-related strains were evaluated against the Ribosomal Database and the phylogenetic tree was built in MEGA7. The evolutionary distances were computed using the Maximum Composite Likelihood method and are in the units of the number of base substitutions per site.

The RAST Server indicated that the most closely related strains of VI4.1 were *Pseudomonas fluorescens* Pf0-1 (205922.3) and *Pseudomonas* sp. GM18, while for VI4T1 these were *Pseudomonas fluorescens* SBW25 and *Pseudomonas extremaustralis* 14-3. Phylogenetic analyses based on the full-length 16S rRNA gene sequences shows the most closely related strains based on the Ribosomal database (Figure 4). *Pseudomonas* sp. VI4.1 clusters with *Pseudomonas fluorescens* (AY538264), while VI4T1 is closely related to the *Pseudomonas veronii* type strain AF064460. The DDH between VI4.1 and its closest genome sequenced relative, *Pseudomonas silesiensis* sp. nov. strain A3T (CP014870), shows a distance of 61.90% (probability DDH >70%, 49%), suggesting that VI4.1 is a new species. The DDH estimate between VI4T1 and *Pseudomonas veronii* 1YdBTEX2 (LT599583) was 87.9%, with a probability of DDH >70% of 95.03%, indicating that VI4T1 belongs to the *Pseudomonas veronii* group.

To gain insights into the genome organisation, the genome assemblies were visualised in Bandage (Figure S4A and Figure S4b). Because of the draft genome completeness, it is still difficult to determine whether one or two chromosomes are present, though both strains contain one plasmid: VI4T1 has a plasmid of 40,201 bp and VI4.1 one of 81,970 bp. Comparative analyses show matches of VI4T1 plasmid with a conjugative plasmid in *Pseudomonas putida* pBI709 and in *Pseudomonas fragi* strain NMC25. The plasmid of VI4.1 shows high similarity with *Pseudomonas fluorescens* strain PC20 plasmid pNAH20, *Pseudomonas putida* NCIB 9816-4, *Pseudomonas frederiksbergensis* AS1, and *Pseudomonas putida* ND6 plasmid pND6-1, all encoding a naphthalene degradation operon (Figure S4). 

**Figure 5:**
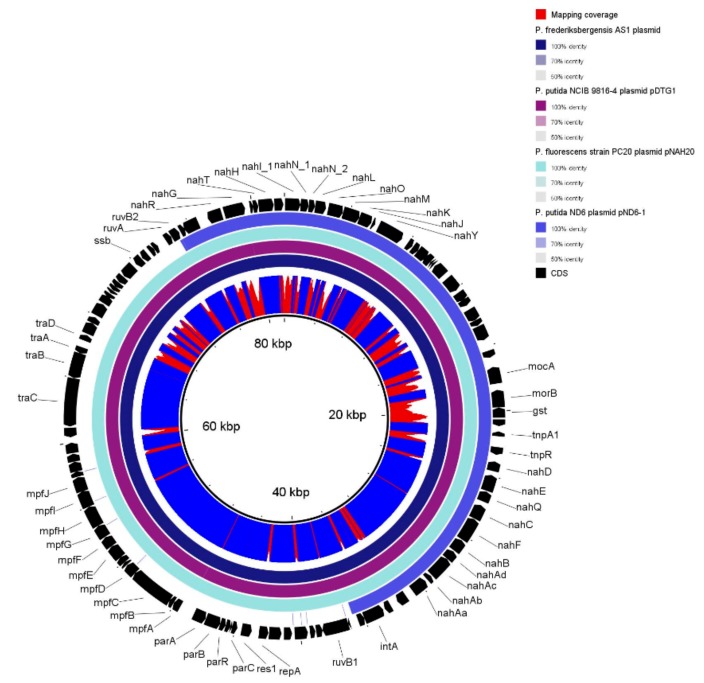
pVI4.1-1 80 kb plasmid encoding a full naphthalene operon, *nahAaAbAcAdBFCED* to convert naphthalene to salicylate, and the lower *nahGTHINLOMKJY* operon to convert salicylate via catechol to tricarboxylic acid (TCA) cycle intermediates.

The 40 kb plasmid of VI4T1 (pVI4T1-1) contains 48 CDSs, while the 81 kb plasmid of VI4.1 (pVI4.1-1) encodes 103 CDSs (Figure S4). pVI4.1-1 has the full aerobic naphthalene degradation operon (*nah1: nahAaAbAcAdBFCED*) which converts naphthalene to salicylaldehyde and salicylate (Figure 5). The enzymes involved are naphthalene 1,2-dioxygenase (*nahAa-d*), which hydroxylates the two-ring compound, and then *cis*-1;2-dihydro-1,2-dihydroxynaphthalene-1,2-dehydrogenase (*nahB*) catalyses the transformation into naphthalene-1,2-diol, with oxygenation by 1,2-dihydroxynaphthalene dioxygenase (*nahC*). Decisive are the actions of the isomerase (*nahD*) and hydratase-aldolase (*nahE*) to obtain salicylaldehyde further dehydrogenated by the enzyme salicylaldehyde (*nahF*). At this point the molecule is metabolized via the catechol cleavage pathway encoded by the lower naphthalene operon (*nah2: nahGTHINLOMKJ*). Salicylate monooxygenase (*nahG*) oxidises salicylate to catechol, and then further transformations are catalysed by the enzymes: ferredoxin (*nahT*), catechol-2, 3-dioxygenase (*nahH*), 2-hydroxymuconic semialdehyde dehydrogenase (*nahI*), hydrolase (*nahN*), hydratase (*nahL*), acetaldehyde dehydrogenase (*nahO*), aldolase (*nahM*), oxalocrotonate decarboxylase (*nahK*), and oxalocrotonate tautomerase (*nahJ*). Furthermore, the typical elements of a conjugative plasmid are found such as the transfer genes (*traA-D*), replication site (*repA*), and a complete type IV secretion system (*mpfABCDEFGHIJ*). These latter components form the secretion machinery including the membrane proteins (VirB6, VirB8, VirB10, VirB9 and VirB7), with three ATPases that form the power unit (VirD4, VirB11, VirB4), and VirB11 and VirB4 are also required for the biogenesis of the T4S pilus. T4SS can mediate in this case the conjugative transfer of the naphthalene operon to other bacteria. Conjugation is an important strategy employed by bacteria to promote bacterial genome plasticity, and to spread a variety of functions, such as the degradation of anthropogenic toxic compounds or the detoxification of heavy metals, but also bacteriocin and toxin production to ward off predators. Having part of the degradation pathways encoded on a plasmid is an interesting property, as the strain can transfer its degradative plasmid via horizontal gene transfer to native soil or plant-associated bacteria, a strategy that has been utilised by our group to clean-up groundwater polluted with toluene and trichloroethylene.

The 40-kb pVI4T1 plasmid does not contain a naphthalene nor aromatic compound degradation operon, though it does contain partial T4SS proteins (MpfA-E), and ParA and B which are bacterial proteins involved in plasmid replication and partitioning, which suggests this was a functional conjugative plasmid but it might have lost some essential genes (Figure S5). For the many CDS annotated as “hypothetical proteins”, we ran a conserved domain search and this revealed the presence of: amidase expression regulating protein, glutathione permease protein (GsiD), histidine/lysine/arginine/ornithine ABC transporter, transposases, cation efflux system (CzcD), transporters (MntH), and components involved in secretion and vesicular transport. It is a subject of speculation as to whether this plasmid is a rudimentary copy of the pVI4.1-1, or a part was not assembled; additional long-read sequencing can confirm this.

### 3.4. Physiological and Biochemical Properties

Strains VI4T1 and VI4.1 are aerobic, heterotrophic, motile, Gram-negative, and non-sporulating strains. The FAME profile of *P. veronii* VI4T1 consisted of 50.39% 16:1 7c/16:1 w6c, 0.15% 18:0 ante/18:2 w6,9c, 0.11% 19:1 w7c/19:1 w6c, 25.51% 18:1 w7c, 0.54% 14:0, 22.6% 16:0, 0.54% 18:0, and 0.15% 18:1 w7c 11-methyl. For VI4.1, the FAME profile was 42.73% 16:1 w7c/16:1 w6c, 0.61% 18:0 ante/18:2 w6,9c, 3.92% 18:1 w7c, 0.55% 10:0 3OH, 4.56% 14:0, 45.39% 16:0, 1.53% 18:1 w9c, and 0.72% 18:0. 

The morphology of stain VI4.1 is smooth, creamy and round, while VI4T1 grows as slightly yellow, smooth and irregular colonies on 869 rich medium. The optimal growth temperature was 30 °C, but the strains showed also good growth at 4 °C. The strains grew preferably on nutrient-rich agar, but they proliferate as well in mineral BH medium and supplemented with diesel oil as the sole carbon source. The strains could grow in 869 medium with NaCl concentrations in the range of 1–4% (w/v) showing salt stress tolerance, and they survived in medium with slight acidic pH, at 5–6.

The ability to capitalize on a variety of carbon sources is an important feature for soil and plant-associated microorganisms. Strain VI4T1 and VI4.1 respired numerous carbon sources as reported in Supplementary Table S1. 

Chemical sensitivity of the strains was also tested with the GEN-III array, and this showed tolerance of VI4T1 to grow at pH 5-6, in the presence of 1% NaCl, 1% sodium lactate, fusidic acid, D-serine, troleandomycin, rifamycin SV, minocycline, lincomycin, niaproof 4, vancomycin, tetrazolium violet, tetrazolium blue, nalidixic acid, potassium tellurite, aztreonam, sodium butyrate, and sodium bromate. Strain VI4.1 was also capable of growing at pH 5-6, up to 4% NaCl, in the presence of 1% sodium lactate, fusidic acid, D-serine, troleandomycin, rifamycin SV, minocycline, lincomycin, guanidine HCl, niaproof 4, vancomycin, tetrazolium violet, tetrazolium blue, nalidixic acid, lithium chloride, potassium tellurite, aztreonam, sodium butyrate, and sodium bromate. 

### 3.5. Degradation of Hydrocarbons

The genome of VI4T1 encodes 45 dioxygenases and 20 monooxygenases, VI4.1 has 50 di- and 27 mono-oxygenases. The dioxygenases comprised naphthalene, biphenyl, phenylpropionate, nitropropane, halobenzoate, and catechol dioxygenase, while for monooxygenases, alkanesulfonate, cyclohexane, alkanal, nitrilotriacetate, and l-ornithine were present. The high number and diversity of dioxygenases and monooxygenases suggests that the strains have versatile metabolic capabilities and hydrocarbon degradation potential. Other strains such *Paraburkholderia aromaticivorans* BN5, recently isolated from an oil polluted site in South Korea, have shown to harbour multiple aromatic ring hydroxylating enzymes, facilitating the use of naphthalene and BTEX as sole nutrient source [46]. For efficient phytoremediation activities, it is important to understand the underlying genetics structures of the many degradation pathways to better assess their activity and use in the field. In the following paragraphs we therefore focus on the bioinformatic prediction of degradation pathways of naphthalene, BTEX, and aliphatic hydrocarbons, and their experimental validation, followed by PGP prediction, motility, and emergent contaminant degradation.

**Figure 6:**
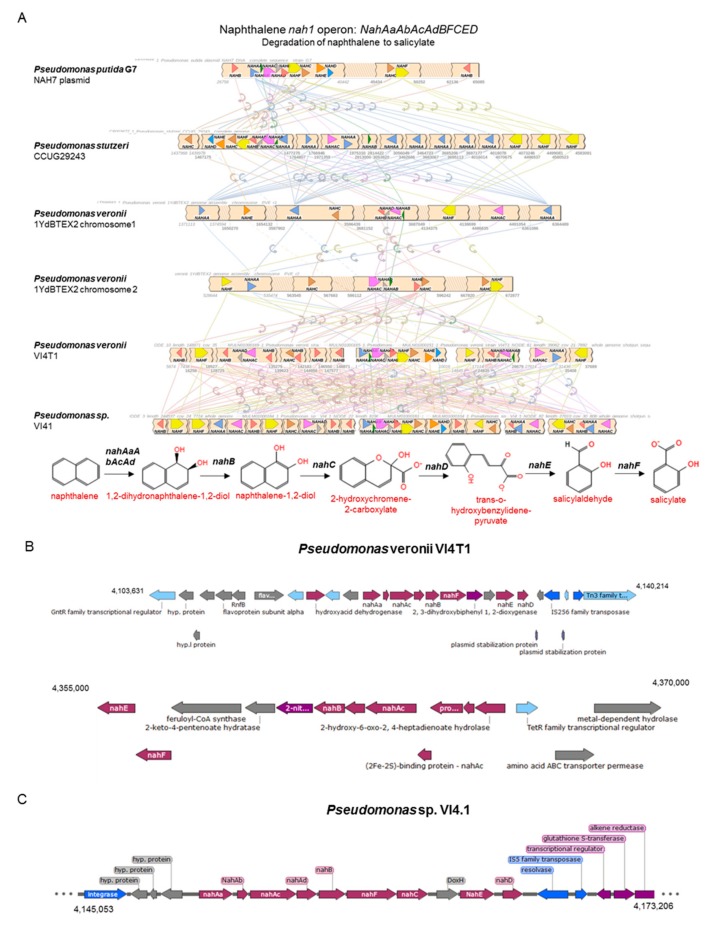
Comparative analyses of the upper naphthalene operon, physical map of the degradation genes and proposed biochemical pathways for naphthalene degradation for strains VI4T1 and VI4.1.

**Figure 7:**
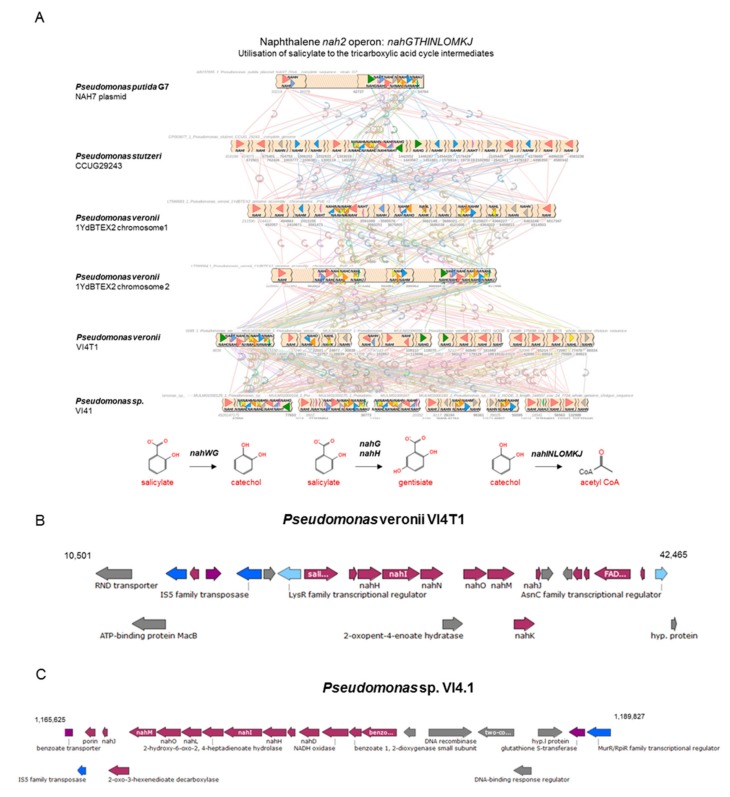
Comparative analyses of the lower naphthalene operon for strains VI4T1 and VI4.1, physical map of the degradation genes, and proposed biochemical pathways for salicylate degradation to catechol or to gentisiate and further breakdown to TCA cycle intermediates.

Previous studies have shown that aerobic naphthalene degradation in *Pseudomonas* spp. can occur via (1*R*,2*S*)-1,2-dihydronaphthalene-1,2-diol, salicylaldehyde, and salicylate, to be converted to catechol and further degraded to tricarboxylic acid (TCA) cycle intermediates. Bioinformatics analyses showed at least two copies of the upper naphthalene operon *nahAaAbAcAdBFCED* for *P. veronii* VI4T1, similar to *P. veronii* YdBTEX2, and one copy for strain VI4.1, encoded on the pVI4.1-1 plasmid (Figure 6). This confirms that strain VI4.1 has obtained the degradation capabilities of naphthalene through plasmid transfer. Also for VI4T1 one copy of the *nah1* operon seems to be plasmid encoded, since plasmid stabilization proteins and transposases are found flanking the operon. For the downstream *nah2* operon (*nahGTHINLOMKJ*) one complete cluster is present in each strain, based salicylate hydroxylase (*nahG*, in green) and 2.3-catechol *nahH* (in blue) as the key enzymes (Figure 7). Organization of this operon between the strains differs considerably, and many of the enzymes are present in multiple copies, such as 2-hydroxymuconic semialdehyde dehydrogenase (*nahI*). The upper and lower pathways are controlled by a LysR family transcriptional regulator (Figure 6 and Figure 7).

Aerobic toluene degradation can occur via five different pathways as described in MetaCyc. Strain VI4.1 harbours the *tomAo12345tomB* operon to degrade toluene via o-cresol (Figure S6). A comparative analysis shows the similarity of the operon structure with strain *Burkholderia cepacia*. Strain VI4.1 has a partial *tbuA1A2BCUVE* monooxygenase operon (missing *tbuU* and *tbuV*) and also a partial *tomABCDE* operon, lacking a homologue for *tomB* (BLAST E-value <0.001, >50% query coverage). Strain VI4T1 does not have homologues to any of the *tom* operons, but seems to use route IV and V. Strain VI4T1 harbours the complete toluene monooxygenase operon *xylMBCXYZL* in two copies to convert toluene and xylene to catechol, and downstream *meta-*cleavage of catechol via *xylEGHFIJK* to actyl-coA which enters the TCA cycle. The operon structures are very similar to the *xyl* operon of the reference strain *Pseudomonas veronii* 1YDBTEX2. The lower catechol ortho-cleavage pathway contains the genes (Figure S7B) catechol 2,3-dioxygenase (*xylE*), 2-hydroxymuconic semialdehyde dehydrogenase (*xylG*), 4-oxalocrotonate isomerase (*xylH*), 4-oxalocrotonate decarboxylase (*xylI*), 2-oxopent-4-enoate hydratase (*xylJ*), 2-oxo-4-hydroxypentanoate aldolase (*xylK*), and an acetaldehyde dehydrogenase (*todI*). The toluene degradation V route (*todC1C2ABCDEF*) to catalyse toluene degradation via toluene-cis-diol is also present in both strains (Figure S8).

Strain VI4T1 encodes also the genes to degrade xylene (*xylBCM*), VI4.1 is lacking the *xylM* homologue (e-value <0.001, coverage of 50%) (Figure S7A). Both strains carry the genes to degrade benzene (*bedC1C2AB*) (Figure S9). Strain VI4T1 has additionally an anthranilate degradation operon *antAaAbAc*, downstream of a tryptophan 2,3-dioxygenase gene, as anthranilate is an important intermediate of tryptophan metabolism [47].

**Figure 8:**
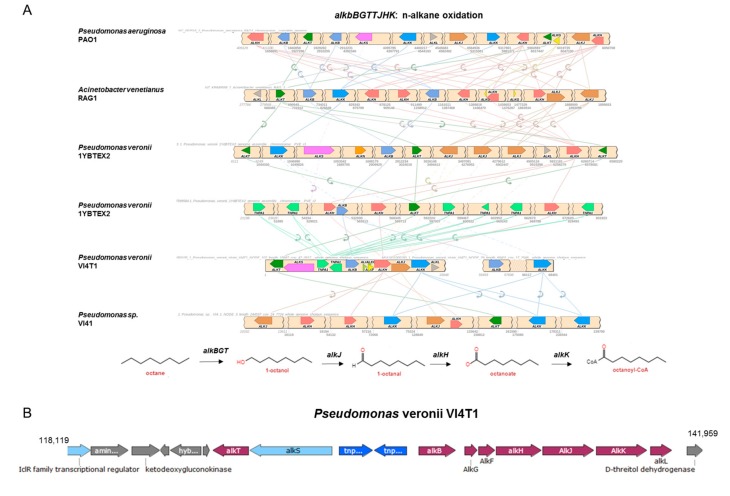
Comparative analyses of the pathways involving alkane-monooxygenase in different *Pseudomonas* strains included *Pseudomonas veronii* VI4T1.

Furthermore, genes encoding for enzymes involved in the linear hydrocarbon degradation were investigated. The complete *alkB* operon was present for VI4T1 (Figure 8) but VI4.1 lacks this alkB operon. The 1-alkane hydrolase subunit (*alkBGT*) introduces molecular oxygen in the terminal carbon atom of the hydrocarbons at the expense of NADH to yield primary alcohols, and these are further catabolized by an octanol dehydrogenase (*alkJ*), an aldehyde dehydrogenase (*alkH*) and a medium-chain acyl-CoA synthetase (*alkK*). Finally, the octanoyl-CoA enters the beta-oxidation-cycle and can be utilized as a carbon and energy source (Figure 8). Instead of the complete *AlkB* operon, VI4.1 encodes a homologue of the two-domain *AlkB* system with rubredoxin and rubredoxin-NAD(+) reductase. Both are essential electron transfer components needed for alkane hydroxylation by AlkB. In fact, AlkB-type alkane hydroxylases fused to rubredoxin protein have been shown to hydroxylate n-alkanes with chain lengths up to C32. VI4.1 and VI4T1 also have a homolog of the cytochrome P450 CYP153 family, this is another type of alkane hydroxylase for the degradation of short- and medium-chain-length *n*-alkanes. Other enzymes important in aliphatic hydrocarbon degradation are the alkanesulfonate genes, of which 11 are present in VI4T1 and 15 in VI4.1. 

**Figure 9:**
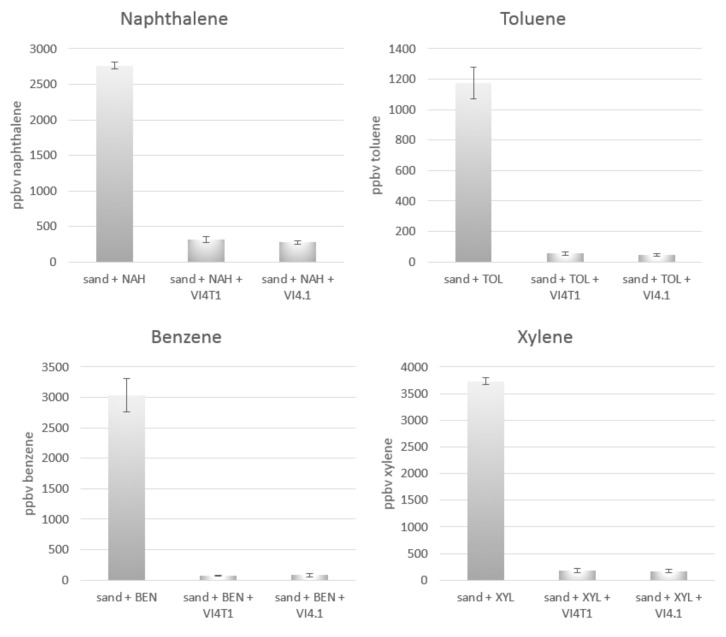
Concentration of naphthalene in the gas phase remaining after seven days of incubation in vials inoculated with *Pseudomonas veronii* VI4T1 and *Pseudomonas* sp. VI4.1. Negative controls are vials containing sterile sand. All vials were spiked in the headspace with 15 µg/L naphthalene or with 3.5 µL/L benzene, toluene, or xylene. 200 µL of bacteria suspension (OD_600_ = 1) were added to the all the vials except for the negative controls. Error bars represent standard error.

To confirm the degradation potential, experimental evidence of aromatic hydrocarbon compound degradation was performed with PTR-TOF-MS. VI4T1 and VI4.1 were both capable of respiring 15 µg/L naphthalene and 3 µg/L each of benzene, toluene, and xylene as sole carbon source within seven days (Figure 9). Metabolites such as salicylic acid or catechol were not identified with PTR-TOF-MS in the headspace. 

### 3.6. Motility and Chemotaxis

Motility of a strain in its environment influences its survival and competence to colonize to find nutrient sources and colonize plant root surfaces [48]. Additionally, motility allows bacteria to move away from high concentrations of toxic compounds. Bacterial motility was experimentally tested in sterile glass tubes previously filled with 869 semi-solid agar medium. Bacteria were inoculated with a straight wire to about half the depth of the medium. Tubes were incubated at 30 °C and observed for bacterial growth at regular intervals (one until seven days). Non-motile bacteria generally grow into the stab-line, while motile bacteria, such as strains VI4T1 and VI4.1, typically grow by differently diffusing through the medium (Figure S10).

Genomic analyses confirmed the presence of genes coding for the flagellar motor complex: *motA*, *motB*, *flhA*, *flhB*, *fliH*, *fliI*, *fliJ*, *fliO*, *flipP*, *fliQ*, *fliR*, *flgB*, *flgC*, *flgG*, *flgH*, *flgI*, *fliE*, *fliF*, *fligA*, *flgD*, *flgN*, *fliK*, *fliS*, *flgE*, *flgK*, *flgL*, *fliC*, *fliD*, *fliG*, *fliM*, *fliN*, *flgJ*, *flhF*, and *fliL*. Furthermore, chemotaxis proteins CheA and CheY are coded. They are involved in the transmission of sensory signals from the chemoreceptors to the flagellar motors. 

### 3.7. Plant Growth Promotion Potential

In vitro tests showed that VI4T1 and VI4.1 scored positive for indole-3-acetate (IAA) production, siderophore production, acetoin, organic acid and phytate mineralization, but negative for ACC-deaminase and nitrogen fixation.

In both genomes, indole-3-acetamide hydrolase was identified converting indole-3-acetamide to IAA. This pathway is operative in several genera of plant-associated bacteria amongst which many *Pseudomonas* spp. [49]. Additionally, genes of the tryptophan biosynthesis gene cluster *trp* were present: *trpD*, *trpG*, t*rpE*, *trpC*, *trpF*, *trpD*, *trpB*, *trpA*. Strain VI4.1 has also the ability to produce IAA via an additional nitrilase-driven pathway converting indole-3-acetonitrile in IAA. The ability to produce siderophores was confirmed by the presence of the pyoverdin pathway genes, 48 genes for 4T1 and 29 for VI4.1. We found also the fundamental genes for the enzymes converting pyruvate in (R)-acetoin, with acetolactate synthase (*bud*B) and diacetyl reductase (*bud*A). Strain VI4.1 also carries the two genes responsible for the (S)-acetoin biosynthesis (*bud*C). The absent production of some organic acids such as citric, lactic, succinic, gluconic, itaconic, acetic, propionic, tartaric, malonic, malic, oxalic, fumaric, and lactobionic acid was confirmed after the genome annotation.

Growth and development of both, plants and bacteria require macro- and micro-nutrients, some of which are available only from external sources like the soil in which they grow. Phosphorous is such an essential macronutrient, and is usually absorbed and utilized by the plants in the form of phosphate. The role of genes as Purple Acid Phosphatases (PAPs) in strain VI4T1 has not yet been fully clarified. Experimental evidence suggests that the enzyme is induced by a lack of phosphate and excreted from bacterial cells, suggesting that it may be involved in phosphate acquisition. The presence of the gene coding for the acid phosphatase confirmed the in vitro potential of strain VI4T1 to convert a phosphate monoester in more bioavailable phosphate in the presence of water. 

No enzymes were detected involved in the biological fixation of atmospheric nitrogen such as *nif* genes or nodulation genes (*nod*), confirming the negative response on the nitrogen fixation test. We did not detect any homologue for 1-aminocyclopropane-1-carboxylate deaminase; that outcome confirmed the results of the corresponding in vitro test. 

The aminopeptidase gene (*pepA*) that is involved in seed germination process was detected in both genomes [50]. The enzyme hydrolyses peptide bonds in tissues as the aleurone layer of the endosperm, the scutellum and the growing tissues of the seedling. The activity of these proteins is reported for several plant species. 

Furthermore, both strains can synthesize jasmonic acid a member of the jasmonate class of plant hormones which is involved in the regulation of quite a number of processes as embryo and generative organs development, ageing, sex determination, seed germination, root growth, adaptation to stress factors [51].

### 3.8. Tolerance to Abiotic Stress

Plants are often exposed to abiotic stresses such as heat, drought, metal pollution, high salinity and acidic pH. In such circumstances, inoculating plants with stress alleviating microorganisms may offer a biological alternative to the existing agrochemicals in agriculture [52]. Both strains carry the *speA* gene involved in the survival of the bacterium in acidic conditions via the arginine-dependent pathway, conferring potential acid resistance by exchanging external arginine for internal agmatine [53]. This effectively consumes protons within the cytoplasm, raising the pH. 

Abiotic stress can create osmotic deficiencies in plant cells. In this context, the presence of trehalose can act as an osmoprotectant, and strains VI4T1 and VI4.1 possess several genes coding for proteins involved in trehalose anabolism, including pathway IV (trehalose synthase converting b-maltose in trehalose), and pathways VI and VII (trehalose synthase producing trehalose from ADP-alpha-D-glucose and glucose) [54]. Trehalose accumulation may act as a biosurfactant and in this way enhancing the biodegradation of hexachlorocyclohexane.

Interestingly, both strains carry genes for salt tolerance including *betA* choline dehydrogenase, alcohol dehydrogenase (*yiaY*, *adhA),* and betaine aldehyde dehydrogenase (*betB*). Glycine betaine (N,N,N-trimethylglycine) is a very efficient osmolyte found in a wide range of bacteria and plants, where it is accumulated at high cytoplasmic concentrations in response to osmotic stress, to act as an osmoprotectant [55].

All organisms living in an aerobic environment are exposed to reactive oxygen species (ROS). Strains VI4T1 and VI4.1 carry genes coding for superoxide dismutases (*sodB*, *sodM*) and catalases (*katE*, *katB*) converting superoxide molecules into oxygen via hydrogen peroxide formation. Furthermore, the genomes encode for glutathione reductase (*gor*) converting two molecules of glutathione in glutathione disulfide with a co-production of reduced glutaredoxin. Oxidative damage to proteins often results in the formation of mixed disulfides within the polypeptides. A primary defence against this damage is mediated by the action of GSH-dependent thiol-disulfide oxidoreductases, also called thioltransferases and best known as glutaredoxins (Grx). These proteins reduce the protein disulfide groups back to their native form. 

Heavy metal transporters are involved in acquisition, metal absorption and detoxification. Both strains possess the genes coding for a cadmium-transporting ATPase and a copper-transporting ATPase (*copA*). Copper is an important element that participates in a high number of enzymatic reactions; in photorespiration, electron transport, in ethylene signalling and many other metabolic processes that have copper-containing enzymes catalysing various reactions. 

Furthermore, an arsenic resistance system was detected: arsenical resistance protein ArsH; HTH ArsR-type DNA-binding domain; arsenical-resistance protein Acr3/Arsenical pump membrane protein/ArsBArsenate reductase. In addition, several cation efflux system proteins are present: cation efflux system protein (CusF), cation efflux system protein (CusA), cation efflux system protein (CusC), Cation efflux system protein (CusB). 

### 3.9. Tolerance to Micro-Pollutants and Emerging Contaminants

During the last decades, there has been an increasing concern about the presence of micro-pollutants including pharmaceutical, human health care products, medicines, endocrine disruptors, fluorinated chemicals, and microplastics that are found in the soil and waters in increasing concentrations [56]. There are 2700 such compounds listed by the Joint Research Centre of the European Commission.

Although the novelty of these investigations translates to a lack of information in prokaryotic databases, the genome sequencing of our two *Pseudomonas* spp. allows us to investigate the presence of genes involved in the degradation of endocrine disruptors. We identified in *Pseudomonas* strain VI4.1 the genes androsterone 3-deydrogenase and testosterone dehydrogenase, respectively involved in the transformation of androsterone and testosterone in the common intermediate metabolite androst-4-ene-3,17-dione [57]. This compound links this catabolic pathway to the one of the androstenedione degradations until mineralization via the propanoyl CoA degradation pathway I or TCA cycle. *Comamonas testosterone* was the first strain characterized for its capability to degrade steroids, and its capacity was compared with *Pseudomonas* spp. after incubation in testosterone sewage in the work of Chen et al. (2016) [57].

Concerning fluorinated chemicals, strain VI4.1 carries the gene coding for the enzyme haloacetate dehalogenase, the only known enzyme that can specifically hydrolyse the carbon-fluorine bond. The enzyme was first described from a *Pseudomonas* spp. in 1965 and since then has been described in multiple bacterial strains. Fluorinated organic compounds have widespread applications as pesticides, herbicides, pharmaceuticals, flame-retardants, refrigerants and foam-blowing agents, and are consequently accumulating in the environment. Only a handful of carbon-fluorine bonds from biological origin are known, such as fluoroacetate, a toxin found in the leaves and seeds of a variety of tropical plants, often in high concentrations. The extreme toxicity of fluoroacetate stems from its similarity to acetate. Fluoroacetate combines with coenzyme A to form fluoroacetyl-CoA, which can substitute for acetyl CoA in the tricarboxylic acid cycle. Fluoroacetyl-CoA reacts with citrate synthase to produce 2-fluorocitrate, a metabolite of which then binds very tightly to aconitase, stopping the cycle.

Interestingly, strain VI4.1 possesses the genes terephthalate 1,2-dioxygenase (*tphA*) and terephthalate dihydrodiol dhydrogenase (*tphA*) converting terephthalate to 3,4 dihydroxybenzoate. Terephthalate is the major precursor for polyester fibres and coatings. Fibres from clothing constitute a major problem in our waterways, also polluting drinking water [58]. Strains able to degrade these fibres are an important resource for the plastics recycling/upcycling industry. In relation to this, *Pseudomonas* VI4T1 carries genes (*bph*A) coding for the enzyme biphenyl dioxygenase subunit A. This enzyme is involved in the first step that synthesizes 2-hydroxy-2,4-pentadienoate and benzoate from biphenyl. Biphenyl is an aromatic hydrocarbon and an important precursor for the production of polychlorinated biphenyls (PCBs).

## 4. Conclusions

Genome analysis of *Pseudomonas veronii* strain VI4T1 and *Pseudomonas* sp. VI4.1 not only revealed the presence of key genes involved in the catabolism of aromatic and aliphatic hydrocarbons but also a diverse set of genes involved in plant growth promotion, stress regulation, and adaptation. Experimental evidence for both hydrocarbon degradation and plant growth promotion confirmed the degradative and versatile metabolic properties. These features make both sequenced strains promising candidates for testing and designing different plant–bacteria systems to perform phytoremediation experiments, and contribute in this way to the development of a cost-effective and eco-friendly method to remediate polluted environments. 

## Supplementary Materials

The following are available online at https://www.mdpi.com/2073-4425/10/6/443/s1, Figure S1: Side and top view of the custom-made sandwich diffusion system to isolate hydrocarbon-degrading bacteria, Figure S2: Multiple sequence alignment of the Rieske unit of the *nahAc* gene of bacterial isolates from the oil-polluted soil in Bóbrka. Reference strain is *Pseudomonas putida* plasmid with *nahAc* encoded on plasmid pAK5, Figure S3: Circular genome views of strains VI4T1 and VI4.1 in addition to the major EGGNOG categories distribution (in %), Figure S4: Visualizations of the SPAdes genome assembly and plasmidSPAdes assemblies of strains VI4T1 and VI4.1. Closed circular plasmids with coverage (in red), are indicated and results of the BLAST searches are shown in the tables, Figure S5: Rudimentary conjugative 40 kb plasmid of pVI4T1-1, showing some T4SS genes (*mpfABCDEH*), and other genes involved in conjugation (*parA*, *parB*, *fmdA*, *hbpA*), Figure S6: Toluene degradation operon to o-cresol for strain VI4.1, Figure S7: (**A**) Comparative analyses of upper toluene/m-xylene degradation operon to generate *m*-toluate. (**B**) *meta*-cleavage pathway of catechol to acetyl Co-A degradation and C. *ortho*-cleavage pathway, Figure S8: Comparative analyses of the toluene V degradation operon (*todABC1C2DFGHI*), and proposed biochemical pathways for toluene degradation to acetyl-CoA, Figure S9: Comparative analyses of the benzene degradation operon (*bedABC1C2D*), and proposed biochemical pathways, Figure S10: Example of positive result to the motility assay, Table S1: The list of carbon sources catabolized by VI4T1 and VI4.1 tested with the GEN-III array (Biolog).
