# Supplementary material for: Genomic Diversity of Two Hydrocarbon-Degrading and Plant Growth-Promoting Pseudomonas Species Isolated from the Oil Field of Bóbrka (Poland)
Source: Genes (Basel). 2019 Jun 11;10(6):443. doi: 10.3390/genes10060443 (PMC6628094; doi:10.3390/genes10060443)
Supplement: Supplementary file 1 [file genes-10-00443-s001.pdf]

# Supplementary Figures and Tables

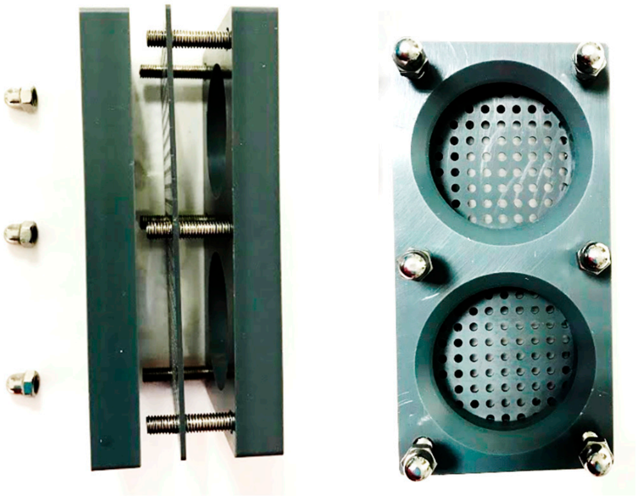

**Figure S1.** Side and top view of the custom-made "sandwich diffusion system" to isolate hydrocarbon degrading bacteria.

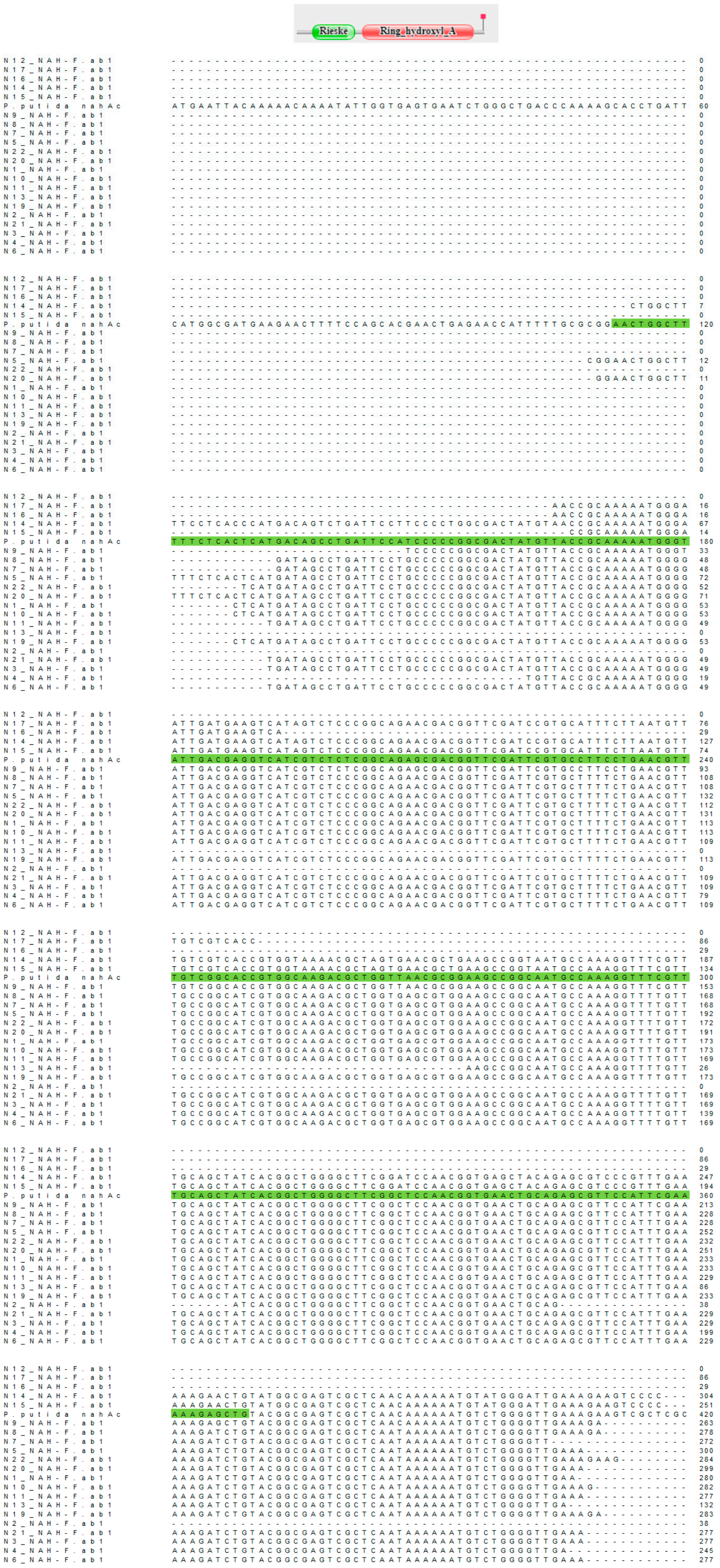


**Figure S2**. Multiple sequence alignment of the Rieske unit of the *nahAc* gene of bacterial isolates from the oil polluted soil in Bóbrka. Reference strain is *Pseudomonas putida* plasmid with *nahAc* encoded on plasmid pAK5.

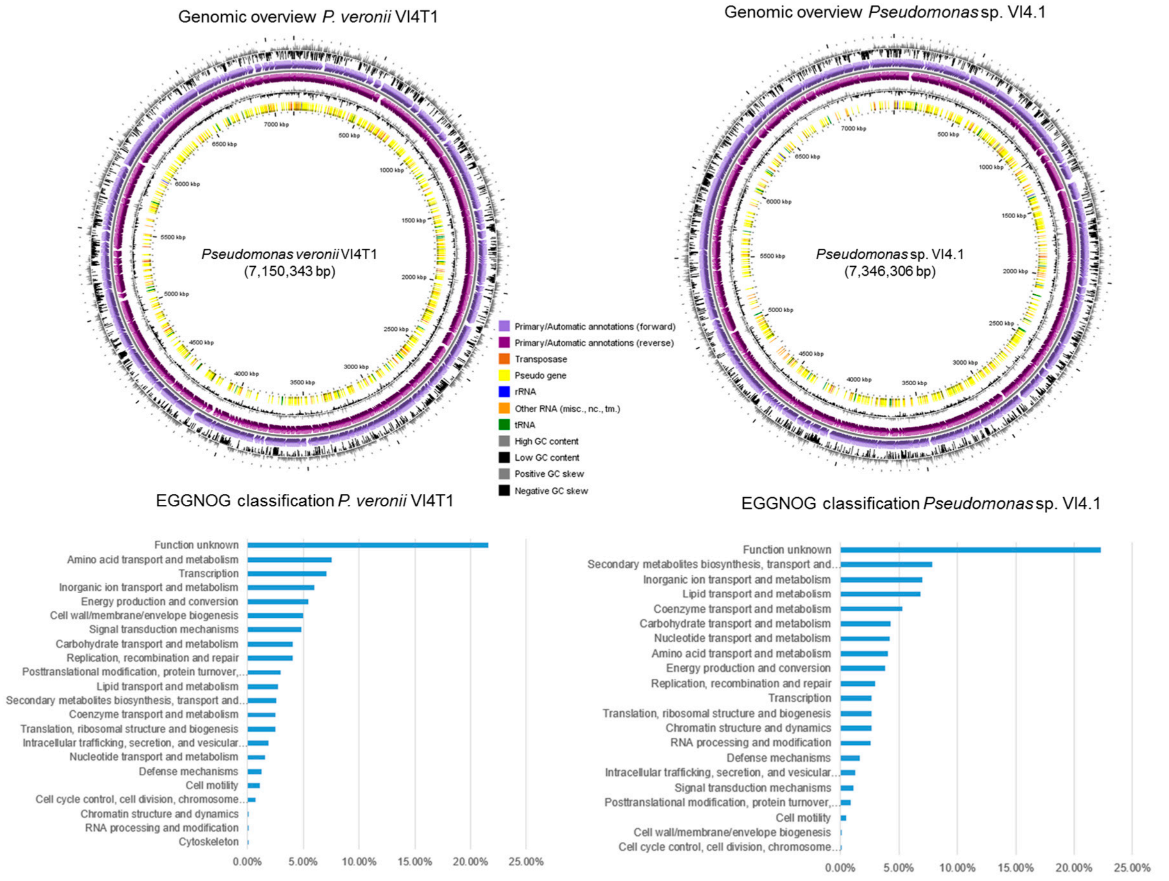


**Figure S3:** Circular genome views of strains VI4T1 and VI4.1 in addition to the major EGGNOG categories distribution (in %).

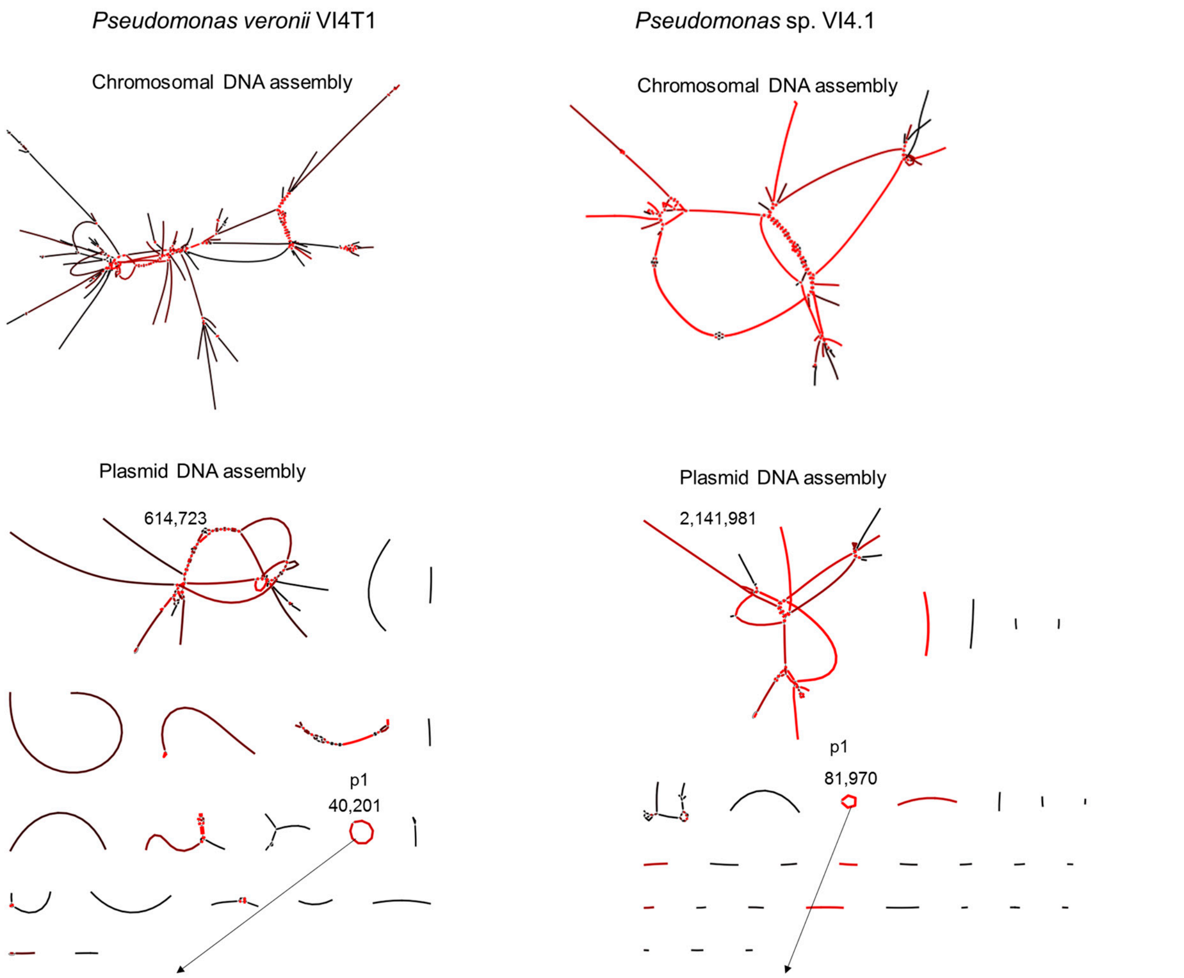


| Best NCBI BLAST hits for p1 | Max Score | Total Score | Query Cover | E value | Per. Ident | Accession |
|---|---|---|---|---|---|---|
| *Pseudomonas fluorescens* strain PF08 chromosome | 13448 | 13602 | 20% | 0 | 95.92% | CP032618.1 |
| *Pseudomonas putida* plasmid pBI709 | 11945 | 11945 | 17% | 0 | 98.17% | AY299015.1 |
| *Pseudomonas brenneri* strain BS2771 chromosome I | 7662 | 17127 | 23% | 0 | 83.89% | LT629800.1 |
| *Pseudomonas fragi* strain NMC25 plasmid | 3733 | 8437 | 24% | 0 | 79.54% | CP021134.1 |

| Best NCBI BLAST hits for p1 | Max Score | Total Score | Query Cover | E value | Per. Ident | Accession |
|---|---|---|---|---|---|---|
| *Pseudomonas fluorescens* strain PC20 plasmid pNAH20 | 87059 | 1.51E+05 | 100% | 0 | 99.99% | AY887963.3 |
| *Pseudomonas putida* NCIB 9816-4 plasmid pDTG1 | 87054 | 1.51E+05 | 100% | 0 | 99.98% | AF491307.2 |
| *Pseudomonas frederiksbergensis* strain AS1 plasmid | 81093 | 1.50E+05 | 100% | 0 | 99.99% | CP018320.1 |
| *Pseudomonas putida* ND6 plasmid pND6-1 | 67429 | 78703 | 51% | 0 | 99.92% | AY208917.2 |

**Figure S4:** Visualizations of the SPAdes genome assembly and plasmidSPAdes assemblies of strains VI4T1 and VI4.1. Closed circular plasmids with coverage (in red), are indicated and results of the BLAST searches are shown in the tables.

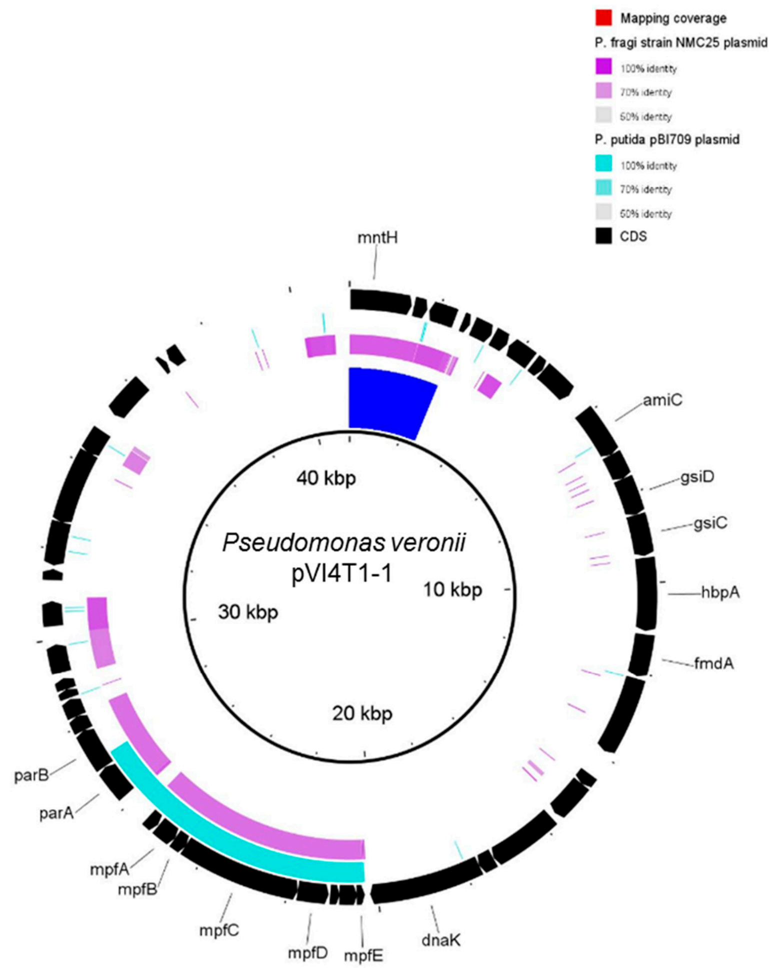


**Figure S5**. Rudimentary conjugative 40 kb plasmid of pVI4T1-1, showing some T4SS genes (*mpfABCDEH*), and other genes involved in conjugation (*parA*, *parB, fmdA*, *hbpA*).

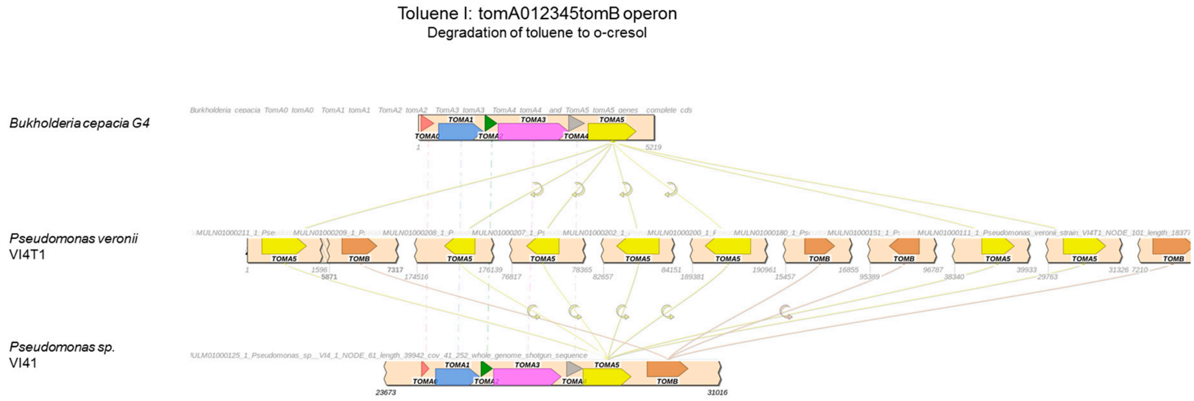


**Figure S6**: Toluene degradation operon to o-cresol.

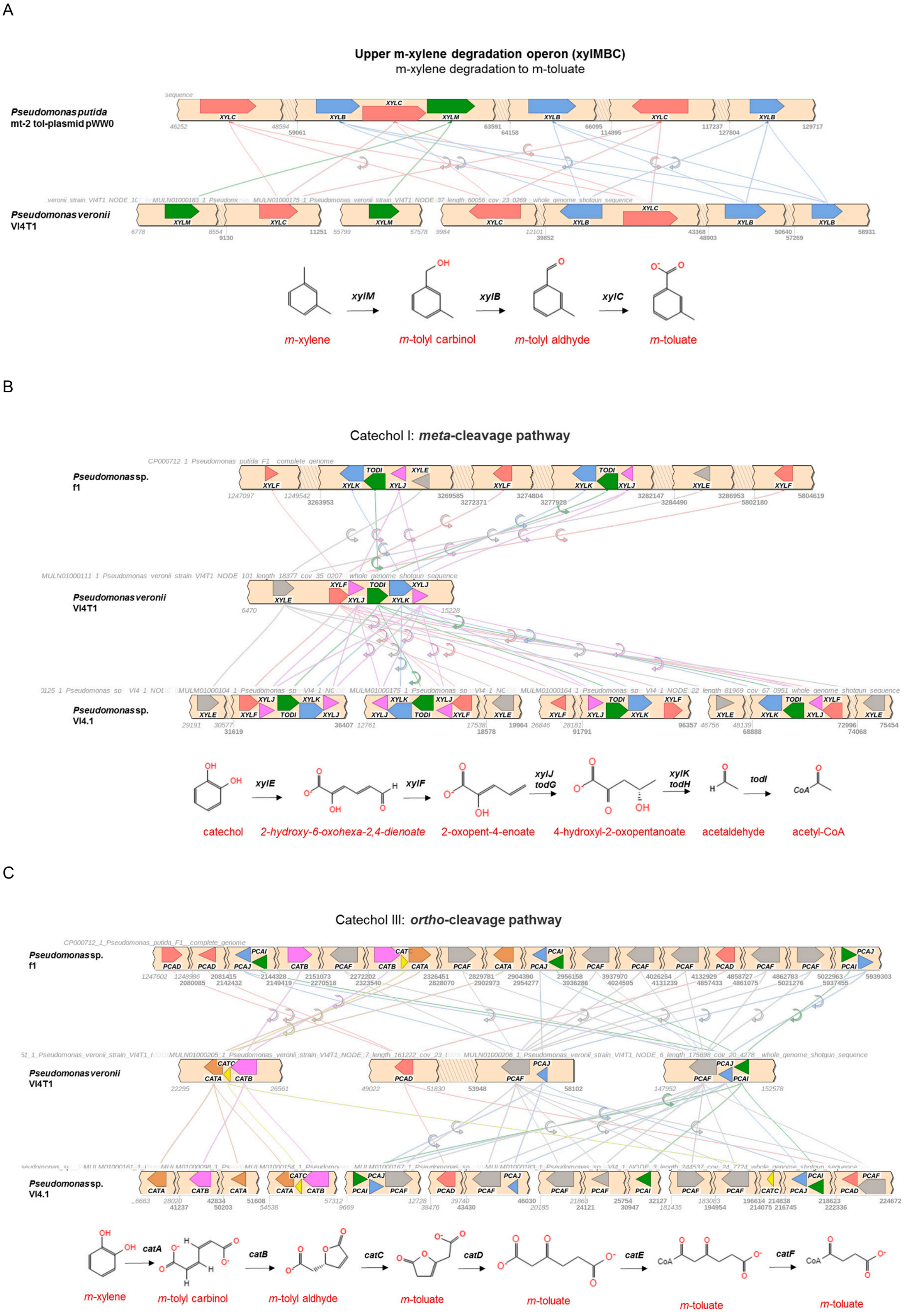


**Figure S7. A.** Comparative analyses of upper toluene/m-xylene degradation operon to generate *m*-toluate. **B.** *meta*-cleavage pathway of catechol to acetyl Co-A degradation and **C.** *ortho*-cleavage pathway.

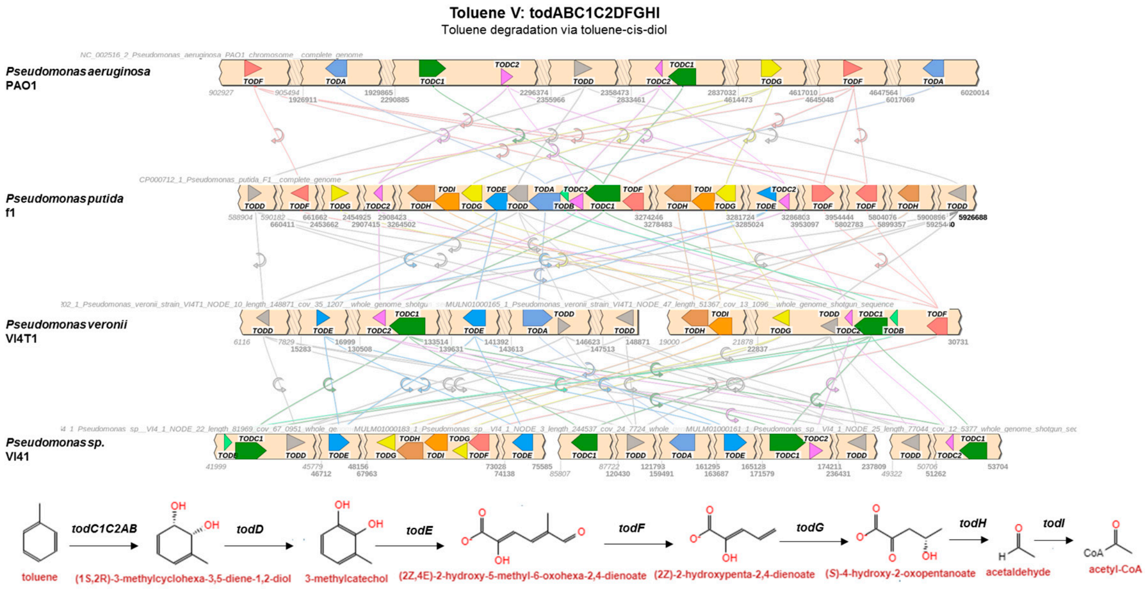


**Figure S8**. Comparative analyses of the toluene V degradation operon (*todABC1C2DFGHI*), and proposed biochemical pathways for toluene degradation to acetyl-CoA.

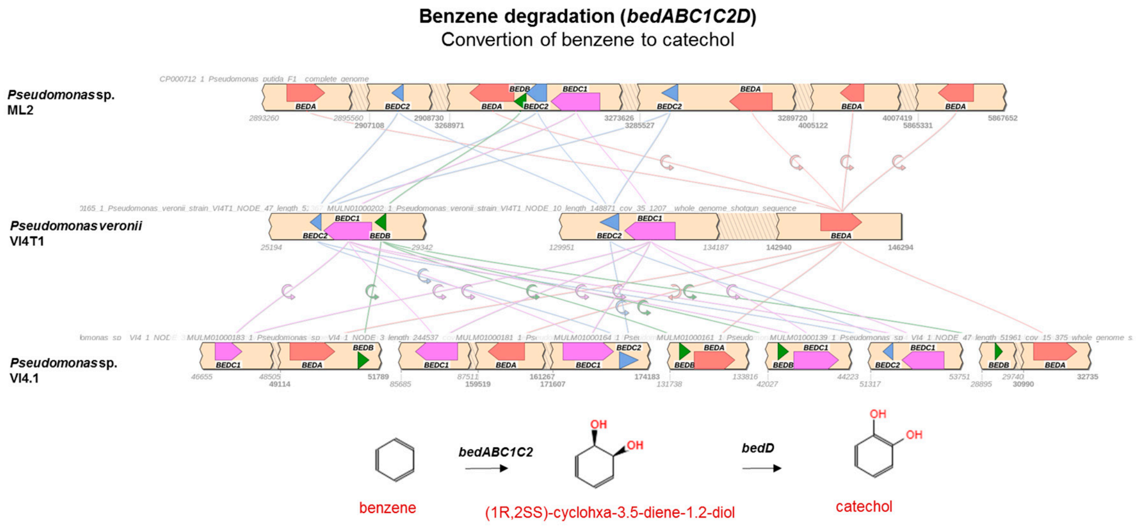


**Figure S9**. Comparative analyses of the benzene degradation operon (*bedABC1C2D*), and proposed biochemical pathways.

**Figure S10.** Example of positive result to the motility assay. Strain 4T1 shows a moderate motility after a week of growth in semi solid rich medium. It spreads beyond the thin stab-line where it was previously inoculated.

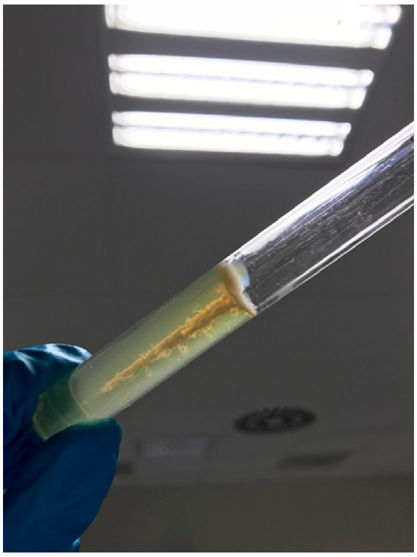

**Table S1: Carbon sources catabolized by VI4T1 and VI4.1 tested with the GEN-III array (Biolog).**

| Carbon sources respired by VI4T1 | Carbon sources respired by VI4.1 |
|---|---|
| D-threalose, sucrose, D-raffinose, α-D-glucose, D-mannose, D-fructose, D-galactose, inosine, D-sorbitol, D-mannitol, D-arabitol, myo-inositol, glycerol, L-alanine, L-arginine, L-aspartic acid, L-glutamic acid, L-histidine, L-pyroglutamic acid, L-serine, D-galacturonic acid, D-gluconic acid, D-glucuronic acid, glucuronamide, mucic acid, quinic acid, D-saccharic acid, methyl pyruvate, L-lactic acid, citric acid, α-keto-glutaric acid, D-malic acid, L-malic acid, Tween 40, γ-amino-butyric acid, α-hydroxy-butyric acid, β-hydroxy-D,L-butyric acid, α-keto-butyric acid and propionic acid | dextrin, D-maltose, D-threalose, D-cellobiose, gentiobiose, sucrose, D-turanose, stachyose, D-raffinose, α-D-lactose, D-melibiose, D-salicin, N-acetyl-D-glucosamine, N-acetyl-D-glucosamine, N-acetyl-β-D-mannosamine, N-acetyl neuraminic acid, α-D-glucose, D-mannose, D-fructose, D-galactose, L-fucose, L-rhamnose, inosine, D-sorbitol, D-mannitol, D-arabitol, myo-inositol, glycerol, gelatin, glycyl-L-proline, L-alanine, L-arginine, L-aspartic acid, L-glutamic acid, L-histidine, L-pyroglutamic acid, L-serine, pectin, D-galacturonic acid, L-galactonic acid lactone, D-gluconic acid, D-glucuronic acid, glucuronamide, mucic acid, quinic acid, D-saccharic acid, p-hydroxy-phenylacetic acid, methyl pyruvate, D-lactic acid methyl ester, L-lactic acid, citric acid, α-keto-glutaric acid, D-malic acid, L-malic acid, bromo-succinic acid, Tween 40, γ-amino-butyric acid, α-hydroxy-butyric acid, β-hydroxy-D,L-butyric acid, α-keto-butyric acid, acetoacetic acid, propionic acid, acetic acid and formic acid |
